# Mid-Term Impact of Kampo Goreisan in Patients with Congestive Heart Failure Receiving Tolvaptan-Incorporated Medical Therapy

**DOI:** 10.3390/jcm15103659

**Published:** 2026-05-10

**Authors:** Yuki Hida, Teruhiko Imamura, Koichiro Kinugawa

**Affiliations:** Second Department of Internal Medicine, University of Toyama, Toyama 930-0194, Japan

**Keywords:** heart failure, diuretics, kampo

## Abstract

**Background**: Despite considerable advances in contemporary pharmacotherapy for heart failure (HF), residual congestion continues to drive adverse outcomes in a substantial proportion of patients. Goreisan, a traditional Kampo herbal formulation, has seen growing clinical application as an adjunct for refractory fluid retention; yet the prognostic implications of sustaining versus withdrawing goreisan among patients on tolvaptan-based regimens have not been adequately characterized. **Methods**: We conducted a retrospective single-center observational analysis enrolling patients with HF who received goreisan alongside tolvaptan-incorporated medical therapy between April 2022 and November 2025. Enrolled patients were classified into a continuation group or a termination group based on their subsequent goreisan treatment course. The primary endpoint was defined as a composite of all-cause death and HF-related hospitalization requiring intravenous diuretic therapy. **Results**: Among 25 patients, 12 maintained goreisan throughout the observation period while 13 underwent treatment termination. Baseline clinical profiles were broadly similar across the two groups. The continuation group exhibited a significantly reduced cumulative incidence of the primary endpoint relative to the termination group (*p* = 0.036). Negative binomial regression revealed a markedly elevated event rate in the termination group (incidence rate ratio 9.27; 95% confidence interval 2.05–42.0; *p* = 0.004), with adverse events demonstrating a pronounced temporal clustering in the immediate post-discontinuation period. **Conclusions**: Among patients with HF and refractory congestion on tolvaptan-incorporated therapy, maintaining goreisan was associated with a trend toward fewer clinical events and longer periods of hemodynamic stability, whereas its withdrawal appeared to be followed by early adverse outcomes. Given the small sample size and observational design, no definitive conclusions can be drawn, and these findings should be regarded strictly as preliminary and hypothesis generating; prospective controlled studies with larger cohorts are needed before any clinical implications can be established.

## 1. Introduction

Heart failure (HF) continues to pose a major challenge to global public health, contributing substantially to morbidity, mortality, and healthcare utilization, and standing among the most frequent indications for hospitalization in older populations across both high-income and low-to-middle-income countries [1,2]. Over recent decades, pharmacological therapy for HF has undergone remarkable progress, with numerous evidence-based therapies now incorporated into contemporary clinical guidelines [1,3,4]. Although these developments have led to meaningful improvements in survival and reductions in hospitalization rates, a considerable proportion of patients still suffer from persistent or recurrent congestion despite optimal guideline-directed medical therapy—a problem that remains incompletely resolved in current practice and carries strong prognostic implications [5].

Loop diuretics have long served as the foundation of decongestion strategies in HF. Nevertheless, their use at high doses—often described as “forced” diuresis—carries the risk of intravascular volume depletion, neurohormonal activation involving the sympathetic and renin–angiotensin systems [6], worsen renal function, and ultimately increase morbidity and mortality [7].

Tolvaptan, a selective vasopressin type 2 receptor antagonist, has been introduced into clinical practice for patients with congestive HF who are refractory to such conventional diuretic therapy [8]. In contrast to loop diuretics, tolvaptan achieves decongestion through aquaresis—selectively inhibiting vasopressin-dependent water reabsorption in the renal collecting duct and thereby facilitating free water elimination with minimal electrolyte disturbance. When used in combination with loop diuretics such as furosemide [9], tolvaptan has been reported to increase urine output, preserve renal function, and reduce HF hospitalizations [10]. However, a subset of patients remains refractory even to such tolvaptan-incorporated medical therapy, suggesting the need for additional therapeutic strategies [11]. In Japan, tolvaptan is approved for the management of fluid retention in patients with HF who show an inadequate response to loop diuretics. Although landmark trials such as EVEREST did not demonstrate a mortality benefit with tolvaptan [8], its combination with loop diuretics has been shown to improve decongestion and preserve renal function in carefully selected patients with diuretic-resistant fluid retention [9,10].

**Figure 1:**
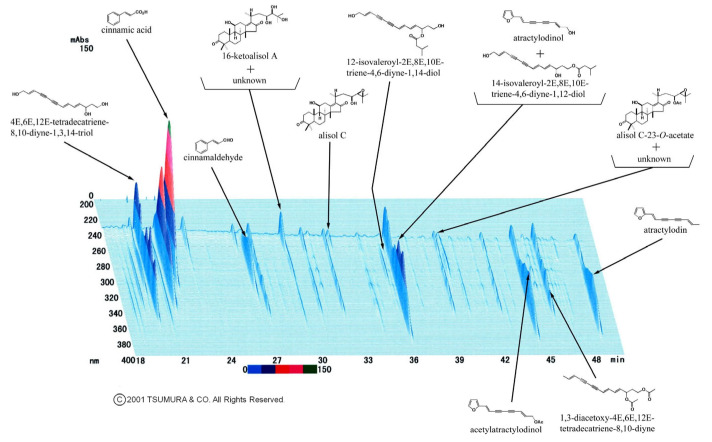
The three-dimensional high-performance liquid chromatography profile of goreisan (reused with permission from Tsumura & Co., Tokyo, Japan). Each peak was identified by comparison with the retention times and ultraviolet spectra of chemically defined standard compounds. The color scale bar (blue to red) represents the absorbance intensity (0–150 mAU).

Kampo medicine has gained growing recognition as adjunctive therapies, particularly among patients whose symptoms remain inadequately controlled despite conventional Western medications (for details, see http://mpdb.nibiohn.go.jp/kconsort/kconsort.html (accessed on 6 May 2026)) [12]. Kampo preparations consist of standardized herbal formulations with a centuries-long history of clinical use in Japan, and they are now formally incorporated into the national healthcare framework under public insurance reimbursement. Among Kampo formulations, goreisan (Tsumura & Co., Tokyo, Japan) has one of the longest histories of clinical application and is composed of five herbal components: Alisma orientale, Polyporus umbellatus, Wolfiporia cocos, Cinnamomum cassia, and Atractylodes lancea (Figure 1) [13]. This formulation is indicated for a range of conditions involving disturbed fluid homeostasis, and has historically been applied to symptoms such as edema, vertigo, cephalgia, and fluid retention-related gastrointestinal complaints. It has been proposed that goreisan may offer a beneficial effect on systemic fluid regulation in patients who remain refractory to maximum doses of conventional diuretic agents, including tolvaptan [14]. Experimental studies have indicated that goreisan may modulate the expression of some aquaporin family members [15,16], key regulators of whole-body water balance, including aquaporin-2 in the renal collecting duct [17]. Through these pathways, goreisan may act through a mechanism distinct from conventional diuretics, targeting water transport at the molecular level rather than primarily increasing urinary sodium excretion.

Despite these mechanistic insights, available real-world data on goreisan use in HF remain confined to short-term reports or limited case series, leaving its mid-term clinical significance largely uncharacterized. Notably, no study has systematically examined what happens clinically when goreisan is continued versus discontinued in patients already established on tolvaptan-based regimens. The present study was therefore designed to evaluate the mid-term clinical consequences of maintaining versus terminating goreisan therapy in patients with congestive HF managed with tolvaptan-incorporated medical treatment.

## 2. Materials and Methods

### 2.1. Patient Selection

Patients with HF who were treated with goreisan between April 2022 and November 2025, together with loop diuretics and tolvaptan, were eligible. Patients who continued goreisan during the study period were assigned to the continuation group, with the day of goreisan initiation defined as day 0. Patients who terminated goreisan were assigned to the termination group, and they were followed from the day of goreisan termination, which was also defined as day 0. In the present cohort, tolvaptan was introduced specifically in patients who demonstrated persistent congestion despite optimization of loop diuretic therapy, consistent with its approved indication in the Japanese clinical setting.

### 2.2. Study Design

This was a retrospective, single-center observational study. All patients were followed from day 0 until the end of the observation period (November 2025). The primary outcome was a composite of all-cause death or HF-related hospitalization. The present study was conducted in accordance with the ethical standards laid down in the 1964 Declaration of Helsinki and its later amendments. Written informed consent was obtained from all participants. The study protocol received approval from the institutional review board of our institution.

### 2.3. Clinical Data Collection

Baseline characteristics, including demographics, comorbidities, laboratory data, echocardiographic findings, and medication use, were collected just before the initiation of goreisan in the continuation group and at the time of goreisan termination in the termination group. During the observation period, all-cause death and HF-related hospitalizations requiring in-hospital management with intravenous diuretics were recorded.

Regarding goreisan administration, all patients received goreisan orally at a standard dose of 7.5 g/day in three divided doses, consistent with the approved prescribing recommendation in Japan. Treatment adherence was assessed at each outpatient visit through medication review and structured patient interview. No formal criteria for clinical response were predefined; rather, the decision to continue or terminate goreisan was made at the discretion of the attending physician, based primarily on patient adherence and overall clinical trajectory. Clinical improvement was assessed informally through changes in body weight, symptom burden, and natriuretic peptide levels during routine follow-up visits.

### 2.4. Statistical Analysis

Significance was defined as a two-tailed *p*-value less than 0.05. The statistical analyses were performed using SPSS Statistics (version 23; IBM Corp., Armonk, NY, USA). Continuous variables were presented as medians with interquartile ranges (25th and 75th percentiles), irrespective of their distribution normality, given the small sample size. Categorical variables were expressed as counts and corresponding percentages.

In the present study, we included two cohorts: the continuation group and the termination group. Continuous variables were compared between the two groups by the Mann-Whitney U test. Categorical variables were compared between the two groups by the Chi-square test or Fisher’s exact test.

In the continuation group, patients were followed following the initiation of goreisan. Patients in the termination group were followed from the time when goreisan was terminated. The primary outcome was death or HF admission.

A swimmer plot was constructed to visualize individual patient follow-up trajectories. Kaplan-Meier analysis and log-rank test were performed to compare the cumulative incidence of the primary outcome between the two groups. The freedom from events was compared between the two groups by Mann-Whitney U test.

Cox proportional hazard ratio regression analysis was performed to investigate the prognostic impact of variables, including the termination of goreisan therapy, on the primary outcome. Variables with *p* < 0.05 in the univariable analysis were included in the multivariable analysis with a forced method. The proportional hazards assumption for the Cox model was assessed using Schoenfeld residuals. For the single covariate (continuation versus termination of goreisan), we examined the correlation between Schoenfeld residuals and (log-transformed) event times.

The negative binomial regression was performed with the number of primary endpoint events as the dependent variable and continuation versus termination of goreisan as the explanatory variable. Follow-up duration was incorporated as an offset term using log-transformed person-years to account for differences in observation time. Incidence rate ratios and 95% confidence intervals between the two groups were calculated.

Given the small sample size and non-normal distribution of continuous variables, non-parametric tests were selected throughout the analysis. Negative binomial regression was chosen over Poisson regression to account for potential overdispersion in event count data. Formal a priori power calculations were not performed, as this study was designed as an exploratory, hypothesis-generating investigation. Accordingly, all statistical findings should be interpreted with caution in the context of limited statistical power, and the results are intended to inform the design of future prospective studies rather than to provide definitive conclusions.

## 3. Results

### 3.1. Baseline Characteristics

During the study period between April 2022 and November 2025, 25 patients initiated goreisan therapy. All patients had received loop diuretics and tolvaptan, as well as guideline-directed medical therapy, for their HF. Of them, 12 patients continued goreisan therapy, whereas 13 patients terminated goreisan therapy at the discretion of the attending physicians (mostly due to patients’ poor compliance). Subsequently, the former patients were assigned to the continuation group, and the latter patients were assigned to the termination group.

Baseline characteristics of both groups were listed in Table 1. Data were not significantly different between the two groups, except for the prescription rate of RAS inhibitors. Fewer patients in the continuation group converted from conventional agents to angiotensin receptor neprilysin inhibitors. Median age was 82 (78, 93) years and median body mass index was 21.7 (20.6, 22.5) kg/m^2^ in the continuation group. Median common logarithm of plasma B-type natriuretic peptide was 2.53 (2.14, 2.67) pg/mL.

The cohort was predominantly elderly, with a median age of 82 years overall. The majority of patients presented with preserved or mildly reduced left ventricular ejection fraction, reflecting a clinically representative population of older adults with advanced congestion refractory to conventional diuretic therapy.

### 3.2. Patient Observation

Following the initiation of goreisan, patients in the continuation group were followed for a median of 408 (374, 562) days. During the period, three patients encountered the primary outcome (one HF admission and three deaths). Following the termination of goreisan, patients in the termination group were followed for a median of 132 (40, 274) days. During the period, seven patients encountered the primary outcome (three HF admissions and five deaths).

**Figure 2:**
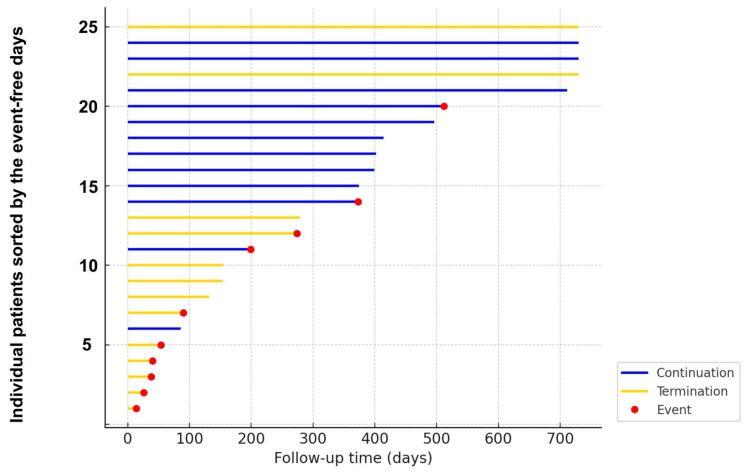
Swimmer plot of event-free follow-up. The event-free durations were lined according to their length. The event-free days appear longer in the continuation group (blue bar) than the termination group (yellow bar). The follow-up appears to be ended by the event (red circle) more in the termination group than the continuation group. The event was defined as a primary outcome, consisting of death or heart failure admission.

A swimmer plot was constructed to visualize individual patient follow-up trajectories (Figure 2). Patients in the continuation group predominantly showed longer event-free follow-up periods, with relatively few early events. In contrast, patients in the termination group frequently experienced early events and shorter event-free follow-up durations, illustrating a consistent pattern of earlier endpoint occurrence following drug termination.

### 3.3. Time-to-Event Analysis

**Figure 3:**
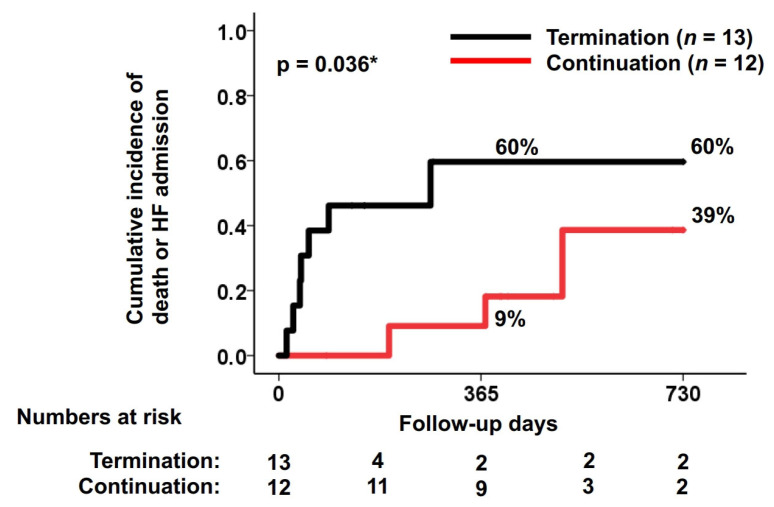
Cumulative incidence of the primary outcome. Cumulative incidence of the primary outcome was significantly lower in the continuation group than the termination group. * *p* < 0.05 by the log-rank test. The primary outcome was defined as a composite of death and heart failure admission.

**Figure 4:**
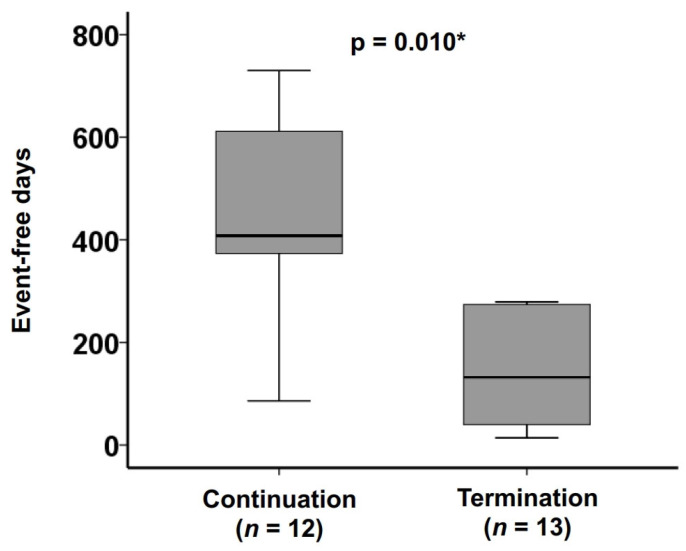
The comparison of event-free duration. The median event-free day was significantly longer in the continuation group than the termination group. * *p* < 0.05 by the Mann-Whitney U test. The event represents the primary outcome.

The cumulative incidence of the primary outcome was significantly lower in the continuation group than the termination group (9% versus 60% at one-year follow-up and 39% versus 60% at two-year follow-up, *p* = 0.036) (Figure 3). Median freedom from the primary outcome was significantly higher in the continuation group than the termination group (408 (74, 562) versus 132 (40, 274) days, *p* = 0.010) (Figure 4).

The termination of goreisan was significantly associated with the primary outcome, with a hazard ratio of 4.00 (95% confidence interval 1.02–16.7, *p* = 0.048) (Table 2). Its association did not reach a statistically significant impact when being adjusted for serum albumin level (hazard ratio 3.34, 95% confidence interval 0.79–14.3, *p* = 0.099).

When we assessed the proportional hazards assumption using Schoenfeld residuals, there was a significant correlation between the residuals for the treatment indicator and log-transformed event times (*p* = 0.037), suggesting some time-dependence of the treatment effect.

### 3.4. Event Rate Analysis

**Figure 5:**
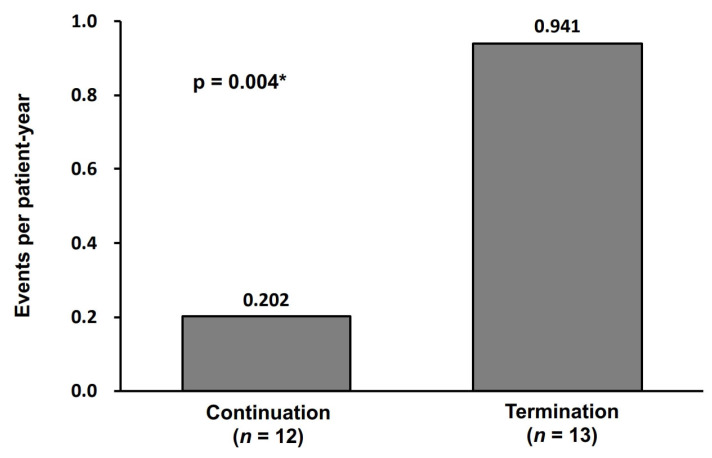
The comparison of event rate. The event rate was significantly higher in the termination group than the continuation group with an incidence rate ratio of 9.27 (95% confidence interval 2.05–42.0, *p* = 0.004). * *p* < 0.05 by the negative binomial regression analysis. The event represents the primary outcome.

The event rate in the continuation group was significantly lower than the termination group (0.202 versus 0.941 events per patient-year) with an incidence rate ratio of 9.27 (95% confidence interval 2.05–42.0, *p* = 0.004) (Figure 5).

## 4. Discussion

### 4.1. Summary of Principal Findings

The present retrospective single-center analysis demonstrated that, among patients with HF and refractory congestion managed with tolvaptan-incorporated therapy, those who maintained goreisan experienced a lower rate of the composite endpoint—all-cause death or HF-related hospitalization—relative to those in whom goreisan was terminated. Adverse events occurred earlier and more frequently following discontinuation, whereas patients sustaining goreisan tended to enjoy prolonged event-free intervals. These observations were made in a clinically complex elderly cohort with multiple comorbidities, consistent with the characteristics of real-world HF populations [18]. Rather than demonstrating treatment efficacy in casual sense, these findings suggest a potential clinical vulnerability associated with treatment discontinuation.

It should be explicitly noted that the present study carries several fundamental methodological constraints. The retrospective, non-randomized design introduces the potential for selection bias and confounding, and the small sample size substantially limits statistical power. These factors collectively preclude causal inference, and the findings must be interpreted strictly within the context of hypothesis generation pending confirmation by prospective randomized investigation.

Furthermore, with only 25 patients across two groups, the present study is substantially underpowered to detect modest treatment effects or to adjust for multiple confounders. The observed differences, while statistically significant, must be interpreted with considerable caution given the limited sample size. These results should therefore serve primarily as a basis for hypothesis generation and for informing the design of future adequately powered studies.

### 4.2. Conceptual Role of Goreisan in Congestion Management

Unlike conventional natriuretic agents, goreisan has traditionally been conceptualized as a modulator of fluid distribution rather than a driver of forced diuresis, with its putative clinical value residing in the preservation of a delicate fluid homeostasis [12,13]. Against this backdrop, the temporal clustering of clinical deterioration following goreisan withdrawal raises the hypothesis that destabilization of fluid regulation may have played a role in precipitating subsequent decompensation. However, the present study did not directly assess objective markers of volume overload, such as serial body weight, natriuretic peptide levels, or imaging findings, thereby precluding definitive mechanistic interpretation.

From a pathophysiological perspective, the pharmacological profile of goreisan may differ fundamentally from conventional natriuretic or aquaretic agents. Whereas loop diuretics promote natriuresis and tolvaptan selectively induces aquaresis via vasopressin V2 receptor antagonism, goreisan has been suggested to influence aquaporin expression, potentially affecting systemic and interstitial water homeostasis [15,16]. Such modulation may theoretically attenuate maladaptive fluid redistribution rather than simply increasing urinary output. In elderly HF patients with impaired vascular compliance and altered capillary permeability, even subtle shifts in fluid compartment balance may precipitate decompensation. Thus, the observed early vulnerability following discontinuation may reflect disruption of a previously stabilized fluid equilibrium. These mechanistic considerations are supported by prior experimental work demonstrating that goreisan modulates aquaporin-2 expression in the renal collecting duct through calcium-sensing receptor activation [17], as well as by studies showing inhibition of aquaporin-4 upregulation in cerebral edema models [16]. Collectively, these findings suggest that goreisan may exert its clinical effects through selective regulation of water channel expression, a mechanism fundamentally distinct from both natriuretic and aquaretic agents currently used in HF management. Whether similar aquaporin-mediated effects operate in the setting of cardiac congestion remains to be directly examined in future translational studies.

### 4.3. Positioning Relative to Conventional Diuretic Strategy

In contemporary HF management, loop diuretics and tolvaptan remain central to congestion control. Loop diuretics induce natriuresis and are effective for rapid decongestion, whereas tolvaptan promotes aquaresis and may preserve renal function in carefully selected patients [9,10]. However, diuretic resistance is increasingly recognized as a major challenge in advanced HF, particularly in patients with repeated hospitalizations and chronic kidney dysfunction [19]. Persistent activation of neurohormonal pathways, including the renin–angiotensin–aldosterone system and sympathetic nervous system, may attenuate the response to loop diuretics over time [5]. In addition, renal venous congestion and progressive renal dysfunction may further impair natriuretic responsiveness, creating a vicious cycle of escalating doses and diminishing efficacy [20].

Although tolvaptan offers an aquaretic mechanism distinct from loop diuretics, its clinical effectiveness may vary in patients with refractory congestion, particularly when systemic hemodynamic reserve is limited [10]. In such settings, therapeutic strategies that do not rely solely on aggressive natriuresis may offer conceptual advantages. A complementary approach that supports overall fluid equilibrium without inducing abrupt intravascular volume shifts may therefore be clinically meaningful, especially in elderly patients with limited physiological reserve. Within this framework, goreisan may be conceptualized not as a substitute for conventional diuretics but as a stabilizing adjunct that modulates systemic water homeostasis in a potentially more gradual manner.

### 4.4. Clinical Relevance in HF with Preserved Ejection Fraction and Elderly Populations

It should be noted that refractory congestion is not exclusive to patients with reduced ejection fraction or significant valvular disease. Even in patients with preserved ejection fraction, diuretic-resistant fluid retention can develop, particularly in elderly individuals with multiple comorbidities.

Although the absence of significant mitral or tricuspid regurgitation and the predominance of preserved ejection fraction in our cohort may appear atypical for advanced systolic HF, these characteristics may still be compatible with a subset of elderly patients with HFpEF who experience persistent congestion. In such patients, congestion may occur despite the absence of overt valvular disease or reduced ejection fraction, potentially reflecting elevated filling pressures, impaired venous compliance, and altered fluid homeostasis.

The clinical relevance of these findings may be particularly pronounced in HF populations characterized by preserved or mildly reduced ejection fraction, advanced age, and multiple comorbidities [21]. In such patients, congestion often develops through a combination of overt volume overload and more subtle alterations in fluid distribution and hemodynamics, making rigid escalation of diuretic therapy challenging [21]. Therapeutic approaches that support stable fluid homeostasis may therefore be clinically meaningful in this setting.

HF with preserved ejection fraction (HFpEF) is now recognized as a multisystem disorder involving microvascular rarefaction, increased myocardial stiffness, and reduced physiological reserve that extends beyond systolic dysfunction alone [22,23]. Congestion in this phenotype may reflect intricate interplay between altered venous compliance, endothelial injury, and dysregulated interstitial fluid dynamics [22,23]. Traditional escalation of loop diuretics may alleviate overt volume overload but may not adequately address these subtler pathophysiological contributors. Furthermore, elderly HFpEF patients often exhibit frailty, sarcopenia, and reduced renal reserve, rendering them particularly susceptible to abrupt hemodynamic shifts [24]. In such individuals, maintaining a stable fluid distribution may be as important as reducing total body volume. Therapeutic strategies that avoid excessive intravascular depletion while supporting systemic water homeostasis may therefore be particularly relevant in this demographic. The predominance of preserved or mildly reduced ejection fraction in our cohort may partially explain why treatment continuity appeared clinically meaningful.

### 4.5. Comparison with Previous Studies

A large-scale propensity score–matched analysis utilizing a Japanese administrative database found no overall reduction in 1-year HF readmission rates with goreisan initiated at hospital discharge, though exploratory subgroup analyses suggested possible benefit in patients with concurrent renal disease [25]. Importantly, that investigation assessed the effect of newly starting goreisan at the point of discharge and did not incorporate detailed phenotypic stratification by left ventricular ejection fraction or underlying HF etiology.

The present study differs fundamentally in its focus: rather than evaluating treatment initiation, it examined the clinical consequences of discontinuing an already established goreisan regimen, predominantly in a cohort with preserved or mildly reduced ejection fraction. The two investigations therefore address distinct but complementary clinical questions—whether beginning goreisan improves prognosis versus whether its withdrawal confers clinical instability. Differences in HF phenotype, treatment context, and background therapy may partly explain the discrepancy between findings.

Of further note, tolvaptan use was limited and contemporary guideline-directed medical therapy was insufficiently characterized in the nationwide study. In contrast, our cohort included patients receiving tolvaptan-incorporated therapy with more consistent contemporary HF management, suggesting a population with more advanced or refractory congestion in whom treatment continuity may be particularly relevant.

### 4.6. Post-Discontinuation Vulnerability and Clinical Implications

The clustering of adverse events shortly after goreisan discontinuation suggests a period of heightened clinical vulnerability. In advanced HF, even modest physiological disturbances may precipitate decompensation [26]. Thus, discontinuation of goreisan may represent not only withdrawal of a supportive therapy but also loss of a stabilizing component within individualized congestion management.

In real-world practice, goreisan is often discontinued due to poor adherence related to powdered formulations or declining general condition. Dosage form characteristics are known to influence medication adherence in vulnerable populations, with powdered formulations being less acceptable in older adults [27]. Accordingly, treatment discontinuation may have occurred more frequently in clinically vulnerable patients. Nevertheless, the temporal association between discontinuation and early adverse events suggests that baseline vulnerability alone may not fully explain subsequent deterioration.

Clinically, discontinuation of goreisan may warrant proactive reassessment of congestion management, including optimization of background diuretic therapy and closer monitoring, as abrupt termination without alternative strategies may increase the risk of early decompensation.

### 4.7. Limitations

Several limitations should be acknowledged. First, the retrospective single-center design, small sample size, absence of randomization, and lack of formal power calculations preclude definitive causal conclusions and limit generalizability. These factors should be considered when interpreting the findings, which are best regarded as exploratory and hypothesis generating. Second, objective congestion markers—such as serial body weight, natriuretic peptide levels, or thoracic imaging—were not systematically assessed, limiting mechanistic interpretation. Third, as only patients without severely reduced ejection fraction were enrolled, the findings may not be applicable to HFrEF populations. Fourth, adherence barriers related to the powdered formulation contributed to treatment discontinuation in some patients, highlighting the need for improved dosage forms and prospective studies on post-discontinuation management. Finally, goreisan is currently approved and reimbursed only within the Japanese national health insurance system, and its limited availability outside Japan restricts the broader applicability of these findings to international clinical settings. Future studies should enroll larger patient cohorts stratified by accepted heart failure phenotypes—including HFrEF, HFmrEF, and HFpEF—to more rigorously evaluate the clinical effects of goreisan across the full spectrum of heart failure presentations.

## 5. Conclusions

The present exploratory study observed that, in a small cohort of patients with HF who had persistent congestion despite tolvaptan-incorporated medical therapy, sustained goreisan use was associated with a trend toward fewer adverse clinical events, while its termination appeared to be temporally associated with early clinical deterioration. Given the small sample size and retrospective nonrandomized design, these findings cannot support definitive conclusions and should be interpreted strictly as hypothesis generating. Whether treatment continuity with goreisan represents a clinically meaningful component of congestion management requires confirmation through larger, prospective, randomized studies stratified by accepted heart failure phenotypes.

## Figures and Tables

**Table 1:** Baseline characteristics.

	Continuation (*n* = 12)	Termination (*n* = 13)	*p*-Value
Demographics			
Age, years	82 (78, 93)	85 (77, 86)	0.73
Male	5 (42%)	6 (46%)	0.57
Body height, cm	151 (142, 165)	159 (150, 165)	0.54
Body weight, kg	51.3 (42.7, 57.4)	49.3 (47.9, 62.9)	0.47
Body mass index, kg/m^2^	21.7 (20.6, 22.5)	22.8 (19.4, 24.2)	0.73
Comorbidity			
Diabetes mellitus	5 (42%)	7 (54%)	0.42
Dyslipidemia	5 (42%)	6 (46%)	0.57
Atrial fibrillation	7 (58%)	11 (85%)	0.14
Coronary artery disease	5 (42%)	6 (46%)	0.57
History of heart failure hospitalization	8 (67%)	9 (69%)	0.89
Laboratory data			
Serum albumin, g/dL	3.2 (2.9, 3.6)	3.3 (2.7, 4.0)	0.78
Hemoglobin, g/dL	11.5 (9.2, 15.0)	10.7 (9.4, 13.4)	0.98
Serum total bilirubin, mg/dL	1.1 (0.8, 1.9)	0.6 (0.4, 1.0)	0.098
Serum sodium, mEq/L	138 (137, 141)	138 (137, 142)	0.30
Serum potassium, mEq/L	4.4 (4.0, 4.6)	4.1 (3.7, 4.7)	0.89
Estimated glomerular filtration rate, mL/min/1.73 m^2^	37.1 (27.6, 50.7)	28.5 (20.4, 38.3)	0.23
Common logarithm of plasma BNP, pg/mL	2.53 (2.14, 2.67)	2.64 (2.25, 2.71)	0.41
Echocardiography data			
Left atrial volume index, mL/m^2^	48 (43, 67)	45 (42, 86)	0.32
Left ventricular end-diastolic diameter, mm	51 (47, 55)	51 (45, 58)	0.53
Left ventricular ejection fraction, %	54 (46, 66)	54 (46, 64)	0.96
Moderate or greater mitral regurgitation	0 (0%)	0 (0%)	-
Moderate or greater tricuspid regurgitation	0 (0%)	0 (0%)	-
Medications			
Beta-blockers	9 (75%)	10 (77%)	0.91
ACE inhibitors or ARB	8 (67%)	3 (23%)	0.036 *
Angiotensin receptor neprilysin inhibitor	4 (33%)	9 (69%)	0.081
Mineralocorticoid receptor antagonist	7 (58%)	10 (77%)	0.32
Sodium-glucose cotransporter 2 inhibitor	7 (58%)	8 (62%)	0.87
Loop diuretics	12 (100%)	13 (100%)	1.0
Dose of furosemide, mg/day	20 (10, 20)	20 (20, 20)	0.41
Vasopressin type 2 receptor antagonist	12 (100%)	13 (100%)	1.0

The whole cohort was divided into two groups according to the continuation or termination of goreisan therapy. Continuous variables were presented as median (25% interquartile, 75% interquartile) and compared between the two groups by the Mann-Whitney U test. Categorical variables were presented as numbers (percentages) and compared between the two groups by the Chi-square test of Fisher’s exact test. * *p* < 0.05. BNP, B-type natriuretic peptide; ACE, angiotensin converting enzyme; ARB, angiotensin receptor blocker.

**Table 2:** Association between the potential variables and the primary outcome.

	Univariable Analysis		Multivariable Analysis	
	Hazard Ratio (95% CI)	*p*-Value	Hazard Ratio (95% CI)	*p*-Value
Continuation of goreisan	4.00 (1.02–16.7)	0.048 *	3.34 (0.79–14.3)	0.099
Age, years	0.99 (0.93–1.08)	0.98		
Atrial fibrillation	1.41 (0.30–6.65)	0.67		
Coronary artery disease	1.79 (0.50–6.43)	0.37		
History of heart failure hospitalization	4.35 (0.21–8.32)	0.17		
Serum albumin, g/dL	0.31 (0.10–0.98)	0.046 *	0.36 (0.12–1.03)	0.056
Hemoglobin, g/dL	1.06 (0.85–1.33)	0.59		
eGFR, mL/min/1.73 m^2^	0.99 (0.96–1.03)	0.81		
Common logarithm of plasma BNP, pg/mL	1.40 (0.26–7.47)	0.70		
Left ventricular ejection fraction, %	1.01 (0.96–1.06)	0.65		

The prognostic impact of potential variables, including the continuation of goreisan, upon the primary outcome, defined as death or heart failure admission, was evaluated by Cox proportional hazard ratio regression analysis. Variables with *p* < 0.05 in the univariable analysis were included in the multivariable analysis with a forced method. * *p* < 0.05. CI, confidence interval; eGFR, estimated glomerular filtration rate; BNP, B-type natriuretic peptide.

## Data Availability

Data are available from the corresponding author upon reasonable request.
